# Spatiotemporal parameters from remote smartphone-based gait analysis are associated with lower extremity functional scale categories

**DOI:** 10.3389/fresc.2023.1189376

**Published:** 2023-07-26

**Authors:** Gabriela Rozanski, Andrew Delgado, David Putrino

**Affiliations:** Department of Rehabilitation and Human Performance, Icahn School of Medicine at Mount Sinai, New York, NY, United States

**Keywords:** mHealth, gait analysis, subjective function, rehabilitation technology, remote patient monitoring

## Abstract

**Objective:**

Self-report tools are recommended in research and clinical practice to capture individual perceptions regarding health status; however, only modest correlations are found with performance-based results. The Lower Extremity Functional Scale (LEFS) is one well-validated measure of impairment affecting physical activities that has been compared with objective tests. More recently, mobile gait assessment software can provide comprehensive motion tracking output from ecologically valid environments, but how this data relates to subjective scales is unknown. Therefore, the association between the LEFS and walking variables remotely collected by a smartphone was explored.

**Methods:**

Proprietary algorithms extracted spatiotemporal parameters detected by a standard integrated inertial measurement unit from 132 subjects enrolled in physical therapy for orthopedic or neurological rehabilitation. Users initiated ambulation recordings and completed questionnaires through the OneStep digital platform. Discrete categories were created based on LEFS score cut-offs and Analysis of Variance was applied to estimate the difference in gait metrics across functional groups (Low-Medium-High).

**Results:**

The main finding of this cross-sectional retrospective study is that remotely-collected biomechanical walking data are significantly associated with individuals' self-evaluated function as defined by LEFS categorization (*n* = 132) and many variables differ between groups. Velocity was found to have the strongest effect size.

**Discussion:**

When patients are classified according to subjective mobility level, there are significant differences in quantitative measures of ambulation analyzed with smartphone-based technology. Capturing real-time information about movement is important to obtain accurate impressions of how individuals perform in daily life while understanding the relationship between enacted activity and relevant clinical outcomes.

## 1. Introduction

As a fundamental aspect of daily life with significant health implications, mobility is routinely assessed in various clinical settings across diverse patient populations to inform therapeutic management (1). Lower extremity injury, neurological disease or musculoskeletal disorders can affect movement in so many different ways that accurate and thorough characterization of deficits is essential (2, 3). An individual's function, here defined as sensorimotor ability to perform everyday tasks (e.g., self-care, housework, ambulating), determines level of independence and long-term outcomes (4). Both objective and subjective functional measures are valuable in providing information about disability or recovery over time, though may reflect distinct domains of physical impairment, activity limitation or participation outlined by the World Health Organization's biopsychosocial (ICF) model/framework (5). Ambulation is a major focus in therapy with wide-ranging implications. Specific features of gait yield valuable insights about underlying physiological processes, reveal divergence from normative patterns such as unequal weight-bearing after stroke and contribute to decision-making for orthopedic conditions (6). In particular, speed has been termed the “sixth vital sign” that predicts future consequences, including falls and hospitalization (2). Asymmetry (i.e., inter-limb imbalance) is also a useful indicator of pathology (7).

Until recently, quantitative biomechanical data could only be obtained with expensive and advanced laboratory equipment, which restricted accessibility for most clinicians. In addition, often limited actionable insights are garnered from these metrics to appreciably change the course of a rehabilitation plan. New motion sensor technology now allows spontaneous monitoring of patients in more ecologically valid environments like home or the community (8–12). The inertial measurement unit (IMU) of smartphones can collect kinematic and spatiotemporal parameters for real-time analysis without the need for costly and inconvenient wearable devices (13–17). This unsupervised, “sensing in context” strategy also avoids potential “Hawthorne/research participant effects” that can influence behavior in the presence of an evaluator (18). While users are aware of recording if software provides prompts to initiate, so-called “background” modes when enabled could further reduce observer bias. Gait assessment performed remotely through mobile applications may be used by therapists to diagnose abnormalities, track disease progression or examine the efficacy of an intervention (19–25). As an emerging approach, continued work is necessary to more widely implement and utilize the information best for therapeutic purposes.

Still, the enacted activity metrics obtained with these novel systems do not represent a subject's full experience, for example pain and other personal factors that impact well-being. Self-report tools, intended to efficiently capture individual perceptions regarding overall health or disease-specific status, are recommended for research trials and routine clinical use (26, 27); however, there is evidence for only modest cross-sectional and longitudinal correlations with performance-based results (28, 29). During rehabilitation (eg. after joint replacement surgery), reliable indicators of progress are particularly important such that the relationship between standard questionnaires and physical ability outcomes in detecting change over time is increasingly studied to inform care practices (30–33). The Lower Extremity Functional Scale (LEFS) is one well-validated measure of impairment affecting physical activities, applicable to a wide spectrum of pathology, that demonstrates excellent reliability and high concordance with related constructs (34, 35). Yet, the relationship to common objective tests evaluating only a single domain of interest through simple time or summary scores is weak (36–38). Few studies incorporate the comprehensive output from motion trackers (39) so more knowledge about how this data relates to subjective scales is needed for appropriate interpretation by health professionals using both results in patient assessment.

The smartphone-based paradigm employed herein provides a unique opportunity for subjects to drive research about free-living mobility outside of controlled settings. Unsupervised movement recording through a readily available consumer device with clear on-screen instructions allows large datasets, otherwise limited by gait lab access, to be accumulated. Before widespread use on a general or at-risk population level, understanding the clinical relevance of this methodology is crucial. Our aim was to determine how remotely captured spatiotemporal parameters relate to perceived lower limb task ability. Therefore, an exploratory analysis was conducted to investigate the association between functional category derived from the LEFS and gait variables, both obtained through a mobile application (OneStep). Since ambulation relies on integrity of the lower body and multiple items on the questionnaire are pertinent to walking, we hypothesized that these measures would have good convergent validity.

## 2. Materials and methods

The de-identified data included in this analysis, from patients working with a physical therapist as part of OneStep's clinical program for at least two weeks during the period of May 2021 to November 2021, were deemed exempt by the Institutional Review Board (Icahn School of Medicine at Mount Sinai, New York, U.S.A.; STUDY-21-01466). While not systematically documented, the most common reason for rehabilitation in this cohort was joint replacement surgery (hip or knee), with a smaller proportion affected by stroke or other neurological condition.

OneStep is an FDA-registered app (available on Google Play and the iOS App Store) that uses a smartphone's integrated IMU (sampling rate 100 Hz) to collect three-dimensional acceleration, angular velocity and magnetic intensity variables. Proprietary algorithms then extract timestamped spatiotemporal parameters based on a user's motion/activity and position of the device (left/right front or back pockets). No calibration is necessary and turns are automatically detected to omit before processing so only straight path walking can be retained. After gait cycle segmentation, the following variables were calculated: cadence, velocity, hip range, base width, step and stride lengths, stance and double support times. Asymmetries of stance, step length and double support were defined as the difference between left and right sides. Validity and reliability of this approach compared to standard quantitative gait evaluation metrics has recently been shown (40–42). Various iPhone and Android devices were utilized in these studies and internal testing has confirmed that different phone models do not impact algorithm performance.

**Figure 1:**
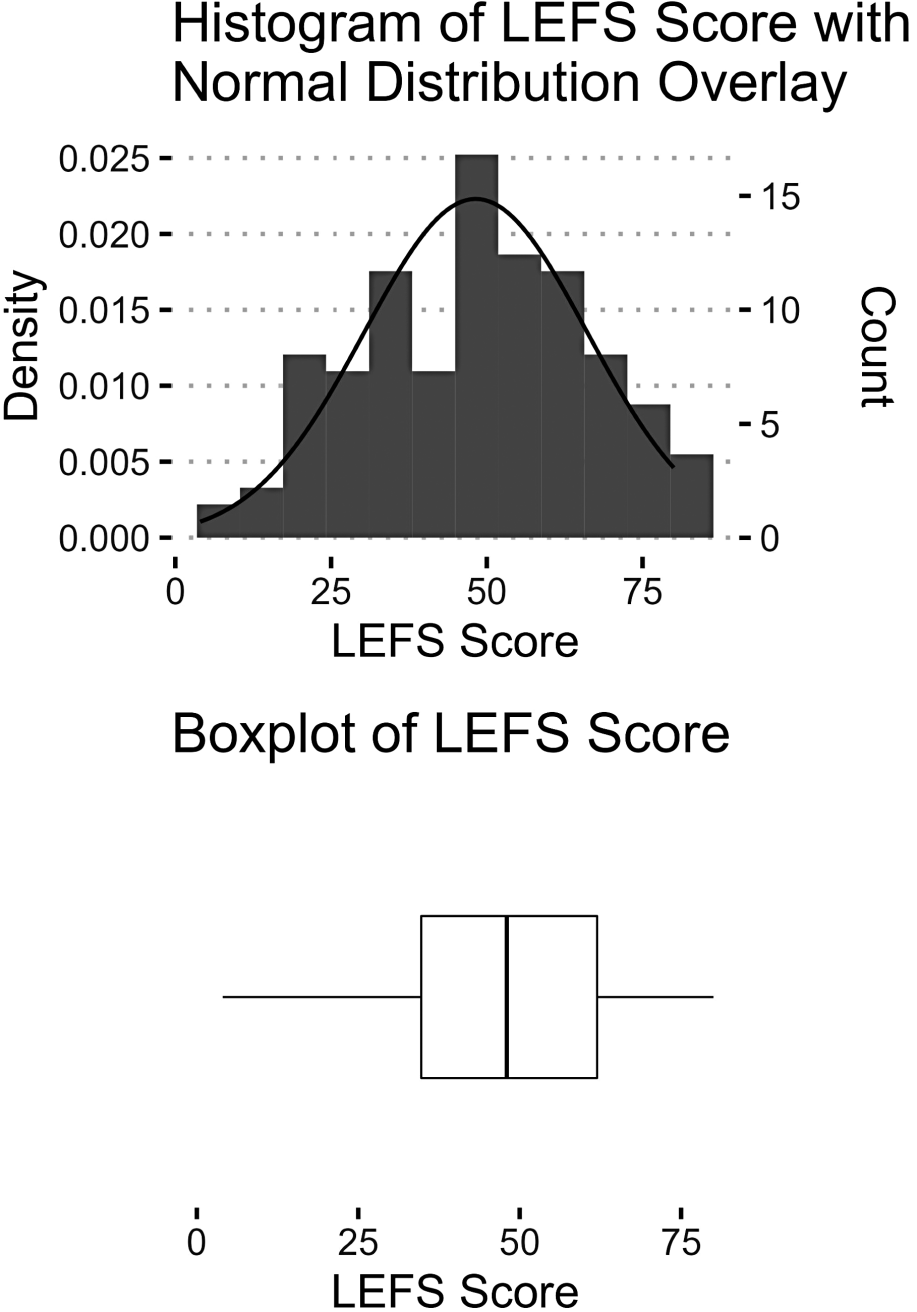
Visualization of LEFS data. Score distribution by histogram (top) and boxplot with maximum, minimum, interquartile range and median values (bottom).

Engagement with the OneStep app is expected to vary across users based on personal factors and individualized exercise plans but all subjects interacted with a similar version of the software. There was no location control for independent home use. While enrolled in physical therapy (duration subject to adherence and treating clinician's rationale), patients were prompted daily to initiate ambulation recordings with the press of a “Start” button in the application (followed by instructions to walk in a straight line for 30–45 s) and notified via pop-up screen to complete the LEFS digital questionnaire on a tri-weekly basis. As a self-assessment tool comprising 20 items that are rated on a five-point difficulty scale, LEFS scores range from zero to 80 and provide subjective measurement about the ability to perform lower body activities (34). Through a functional staging approach reflective of the instrument's structure, psychometric properties and distribution of normative values, discrete categories were created (43–45). Based on numerical value of the questionnaire's response levels, cut-off points at 20, 40, and 60 delineated LEFS groups consistent with a total if the same option was assigned for all answers [*quite a bit* (1), *moderate* (2), and *a little bit of difficulty* (3), respectively]. To achieve size uniformity accounting for skewness of this dataset (see Figure 1), the lower two categories (*a little bit* or *no difficulty* with the activity) were grouped together, which resulted in three function ranks facilitating efficient clinical interpretation according to tallied sum: Low (0–20), Medium (21–40), High (41–80). Previous work has similarly simplified the original LEFS rating scale to ease use (43).

Gait and LEFS data were analyzed on the participant level if both records could be obtained within a one week window to ensure appropriate temporal alignment. This accounted for variation in timing of app usage. When multiple ambulatory sessions met this criterion, only the earliest instance was included to minimize confounding factors from recovery over time and walking bouts were truncated at 60 s to avoid potential effects of fatigue. Due to the proprietary and sensitive health-related nature of the data source, there was limited personal information available to the study team (e.g., 48% of the sample were missing gender identifiers since this was not a required field for enrollment with OneStep services) so these characteristics were not incorporated into the analysis. Unilateral outputs (ie. stance, step length) were specified as high or low after comparison. Given the mixed clinical sample and variability in dominance patterns (i.e., the weaker or post-surgical limb may not always present as such), this simple convention by relative magnitude of values was chosen to avoid over-interpreting results.

Descriptive statistics were reported on all spatiotemporal parameters. Distributions were examined for normality using histograms and Q-Q plots. Continuous and categorical metrics are presented as mean (standard deviation) and count (frequency/percentage), respectively. Hypothesis tests were two-tailed with *p* < .05 considered statistically significant. This being an exploratory investigation, average standardized mean difference (aSMD) was used as the measure of magnitude between groups to provide an overall estimate (Cohen's d for each unique comparisons of “Low”, “Medium”, and “High” LEFS categories); effect sizes were classified as Small (0.2–0.5), Medium (0.5–0.8), and Large (≥0.8) (46). Due to multiplicity concerns, all *p*-values were adjusted using the Benjamini-Hochberg false discovery rate procedure since Bonferroni correction is too conservative if many tests are performed (47). For estimating how gait variables differ across LEFS mobility categories, the One-Way Analysis of Variance (ANOVA) or Kruskal Wallis One-Way ANOVA (KW-ANOVA) was used, according to normality. The non-normally distributed outcomes analyzed by KW-ANOVA were cadence variability, double support time and each asymmetry measure (stance, double support, step length). Only means (standard deviation) are presented for consistency. Analyses were performed in R software v4.0.2 (R Foundation for Statistical Computing, Vienna, Austria) (48, 49) with the following packages: “tableone” (50), “lubridate” (51), “summarytools” (52), “Hmisc” (53), and “tidyverse” (54).

## 3. Results

There were 132 OneStep users included in this analysis. From the available data for gender (*n* = 87, 65.9%), females represented 74.7%. Age was recorded in 43.2% cases (*n* = 57), comprising as follows: 8.8% less than 30 years old, 17.5% 30 to 45, 5.3% 45 to 60 and 68.4% over 60 years old. A diagnosis was known for 63.6% (*n* = 84) of the sample. The highest share were post-surgical, presenting with total hip replacement (*n* = 33, 39.3%), total knee replacement (*n* = 18, 21.4%) or both (*n* = 3, 3.6%). Another 29.8% (*n* = 25) had an orthopedic condition (i.e., musculoskeletal pain/injury) affecting the lower body and 6.0% (*n* = 5) could be classified as neurological. Overall, the mean LEFS score was 48.2 (17.9) with an even distribution across functional categories of Low (0–40, *n* = 44, 33.3%), Medium (41–60, *n* = 49, 37.1%) and High (61–80, *n* = 39, 29.6%) (Figure 1). As shown in Table 1, the largest observed effect size was from velocity (aSMD = 1.044), followed by cadence (aSMD = 0.971), step length-low (aSMD = 0.888) and stride length (aSMD = 0.886). Most of the spatiotemporal variables had medium to large effect sizes; 5 of the 14 examined (35.7%) were small: cadence variability, base width, step length asymmetry, stance asymmetry, and double support asymmetry. The ANOVA analyses found significant differences between LEFS groups after Bejamini-Hochberg correction for all outcomes except cadence variability, base width, and stance asymmetry (Table 2). Means were significantly larger for cadence, velocity, stride length, hip range and step lengths in the High LEFS category while the Low group had higher stance times, double support and asymmetry measures (step length, double support).

**Table 1:** Spatiotemporal variables and zero-order effects between categories of LEFS.

Gait parameter	LEFS group				*p* value	aSMD^a^
	Total (*n* = 132)	Low (*n* = 44)	Medium (*n* = 49)	High (*n* = 39)		
Cadence (steps/min)	99.48 (15.32)	90.06 (12.95)	99.97 (13.34)	109.48 (13.73)	<.001	0.971
Cadence variability (steps/min)	2.27 (1.11)	2.43 (1.06)	2.12 (1.13)	2.27 (1.15)	.411	0.186
Velocity (km/h)	3.27 (1.12)	2.55 (0.80)	3.28 (0.88)	4.06 (1.17)	<.001	1.044
Stride length (cm)	106.97 (23.78)	93.17 (20.12)	107.78 (19.99)	121.51 (23.42)	<.001	0.886
Double support (percent of gait cycle)	32.10 (5.80)	35.38 (5.55)	31.73 (5.24)	28.87 (4.81)	<.001	0.832
Hip range (degrees)	28.91 (12.15)	25.06 (8.29)	27.85 (9.86)	34.58 (16.02)	.001	0.520
Base width (cm)	18.92 (4.03)	20.05 (3.88)	18.60 (3.92)	18.03 (4.14)	.057	0.339
Step length asymmetry (difference in percent of stride length)	3.37 (3.53)	4.30 (3.82)	2.50 (2.53)	3.40 (4.03)	.048	0.350
Stance asymmetry (difference in percent of gait cycle)	2.92 (2.96)	3.65 (3.73)	2.66 (2.40)	2.41 (2.50)	.120	0.271
Double support asymmetry (difference in percent of gait cycle)	1.40 (1.42)	1.56 (1.05)	1.36 (1.47)	1.26 (1.70)	.608	0.146
Stance-high^b^ (percent of gait cycle)	67.52 (3.76)	69.53 (3.82)	67.20 (3.40)	65.64 (3.03)	<.001	0.751
Stance-low^b^ (percent of gait cycle)	64.59 (2.67)	65.87 (2.80)	64.53 (2.25)	63.22 (2.35)	<.001	0.705
Step length-high^b^ (cm)	55.19 (12.17)	48.58 (10.62)	55.11 (9.79)	62.76 (12.36)	<.001	0.852
step length-low^b^ (cm)	51.78 (11.94)	44.59 (9.85)	52.67 (10.31)	58.76 (11.70)	<.001	0.888

Values are mean (standard deviation).

aSMD, average standardized mean difference.

^a^ Effect sizes were interpreted as Small = 0.2–0.5, Medium = 0.5–0.8, and Large = 0.8 or higher.

^b^ Designated according to the relatively longer/larger or shorter/smaller side.

**Table 2:** Analysis of variance across LEFS groups.

Gait parameter	Statistic	df	*p*_adj_
Cadence (steps/min)	22.00	2	<.001^a^
Cadence variability (steps/min)	3.38	2	0.189
Velocity (km/h)	26.13	2	<.001^a^
Stride length (cm)	18.71	2	<.001^a^
Double support (percent of gait cycle)	27.66	2	<.001^a^
Hip range (degrees)	7.29	2	0.002^a^
base width (cm)	2.93	2	0.066
Step length asymmetry (difference in percent of stride length)	9.05	2	0.015^a^
Stance asymmetry (difference in percent of gait cycle)	3.33	2	0.189
Double support asymmetry (difference in percent of gait cycle)	6.86	2	0.041^a^
Stance-high^b^ (percent of gait cycle)	13.47	2	<.001^a^
Stance-low^b^ (percent of gait cycle)	11.81	2	<.001^a^
Step length-high^b^ (cm)	17.59	2	<.001^a^
Step length-low^b^ (cm)	18.77	2	<.001^a^

^a^ Asterisks indicate significance with Bejamini-Hochberg correction.

^b^ Designated according to the relatively longer/larger or shorter/smaller side.

## 4. Discussion

The results of this cross-sectional retrospective analysis suggest that remotely-collected gait metrics are significantly associated with subjects’ category of function based on LEFS scores. While the relationship between objective outcomes and self-assessment measures of lower extremity performance has previously been investigated, to our knowledge no other studies have incorporated spatiotemporal parameters from mobile devices obtained outside the clinic or laboratory setting for this purpose. Mobility level classification (Low, Medium, High) according to the LEFS is also a novel approach, but the cut-off values chosen should be validated through prospective trial design. As a proof-of-concept, clinically-relevant data in various forms (i.e., kinematics, questionnaire) can be captured via (OneStep) smartphone application on a relatively large scale. These findings may have practical informative value for the (tele)rehabilitation context, owing to the substantial sample size and broad inclusion criteria. Engagement with e-health technologies by older adults is encouraging for increased uptake and acceptance to access purported benefits (55). Yet, the lack of specific clinical characteristics to examine as influencing factors greatly limits a multivariate approach and precise interpretation about the effects observed. This study could be less applicable to neurological conditions, which were underrepresented in the user cohort. Further work is needed to determine whether this convergent validity holds in specific situations (e.g., recovery stage, level of severity). Age and surgical history were shown to impact LEFS scores in a healthy reference population (45), though only modestly compared with the magnitudes of difference between groups seen here. By calculated aSMD, velocity was the strongest predictor for functional categorization, like an earlier report similarly demonstrated using another subjective tool (28) and in line with the significant body of literature on this robust metric (3). A possible explanation is that patients seem to interpret difficulty as time to complete a task along with pain and exertion (36), which plausibly affect the speed of an activity too. Weaker associations were found for the asymmetry variables, suggesting certain aspects of movement quality are not considered very important in self-evaluation, despite therapy programs commonly addressing unilateral impairment (e.g., hip/knee arthroplasty or post-stroke hemiplegia). Because interlimb imbalances have potential negative consequences (56), accurate detection provides additional insights for condition management; however, focusing on more easily observable biomechanical features (i.e., cadence, stride length) may confer a greater sense of functional ability. Symmetry metrics are also relevant to higher level tasks that challenge stability (e.g., single leg stance) so other perceptive domains such as balance confidence could be interesting to investigate.

Understanding the basis of individual perceptions can be particularly valuable when change is tracked over time. It is unclear whether the LEFS and walking measurements would continue to relate longitudinally since responsiveness may differ between outcomes (30, 57, 58). Therefore, collecting data to address multiple domains of physical performance remains appropriate for a comprehensive impression about recovery, which aligns with current research and clinical practice recommendations (26, 27). To efficiently streamline workflows or protocols though, individual measures should be compared since redundancies in analytic value potentially exist. The formation of patient subgroups has plausible utility as well, perhaps in order to adapt care accordingly and triage when resources are limited, considering prior evidence that post-operative improvements depend on self-reported function (59). Higher LEFS scores may indicate less progress will be achieved throughout rehabilitation due to possible ceiling effects and physiological adaptation constraints. Expectation and other cognitive components are also likely important factors. In this case, clinicians could incorporate supplemental standardized tests or quantitative gait analysis revealing residual impairments for a more sensitive metric of treatment efficacy and to support decision-making when targeted interventions are applied. Additional insights might be gained from obtaining information about daily life activity if an individuals' own evaluation does not reflect actual behavior. Ecologically valid task items and parameters are necessary to determine whether there is a performance gap between hypothetical capacity and enacted function in “real world” environments. Future research to explore how objective and subjective assessment methods each operate in specific contexts or under particular conditions will be facilitated by remote monitoring technology like the OneStep application for optimal usage of these separate but associated tools within the evolving telehealth care system.

## Data Availability

The raw data supporting the conclusions of this article will be made available by the authors, without undue reservation.
